# Multimodal prehabilitation enhances perioperative outcomes in gastric cancer patients: a single-center randomized controlled trial

**DOI:** 10.3389/fnut.2025.1676180

**Published:** 2026-02-03

**Authors:** Guang-Chuan Mu, Yuan-Hui Tu, Hai-Lun Xie, Si-Yu Liu, Kui Jia, Min-Ying He, Ye-Yang Chen, Jun-Qiang Chen

**Affiliations:** 1 Department of Gastrointestinal Surgery, The First Affiliated Hospital of Guangxi Medical University, Guangxi Medical University, Nanning, Guangxi, China; 2 Guangxi Key Laboratory of Enhanced Recovery After Surgery for Gastrointestinal Cancer, Guangxi Medical University, Nanning, Guangxi, China; 3 Guangxi Clinical Research Center for Enhanced Recovery After Surgery, Guangxi Medical University, Nanning, Guangxi, China; 4 Guangxi Zhuang Autonomous Region Engineering Research Center for Artificial Intelligence Analysis of Multimodal Tumor Images, Guangxi Medical University, Nanning, Guangxi, China; 5 Department of Clinical Nutrition, The First Affiliated Hospital of Guangxi Medical University, Guangxi Medical University, Nanning, Guangxi, China; 6 Department of Breast and Thyroid Surgery, The First People’ Hospital of Yulin, Yulin, China

**Keywords:** multimodal prehabilitation, gastric cancer, perioperative outcomes, functional capacity, quality of life

## Abstract

**Introduction:**

Multimodal prehabilitation, integrating exercise, nutrition, and psychological support, has shown value in perioperative care for gastrointestinal cancers, but its efficacy—especially as a short-course intervention tailored to gastric cancer’s need for timely surgery—remains insufficiently validated.

**Methods:**

Eligible patients undergoing radical gastrectomy received either a 1-week multimodal prehabilitation program plus standard perioperative care (prehabilitation group) or standard perioperative care alone (control group). Primary endpoint was the 30-day postoperative complication rate; secondary endpoints included functional capacity, patient-reported outcomes, recovery metrics, and hospital stay.

**Results:**

Recruitment was conducted from July 2022 to July 2024. A total of 150 patients were randomized, with 131 completing the trial (66 in the prehabilitation group, 65 in the control group). Baseline demographics (gender, age, education level) and clinical characteristics (comorbidities, TNM staging, surgical details) were comparable between groups (all *p* > 0.05). For the primary endpoint, the prehabilitation group had a significantly lower 30-day overall complication rate (9.1% vs. 26.2%, *p* = 0.010), with the largest reduction in pulmonary infections (6.1% vs. 21.5%, *p* < 0.05). For secondary endpoints, the prehabilitation group showed earlier time to first flatus (58.32 ± 26.82 vs. 89.55 ± 26.14 h, *p* < 0.001), shorter interval to oral intake (48.43 ± 31.98 vs. 105.85 ± 57.36 h, *p* < 0.001), reduced time to ambulation (25.14 ± 10.63 vs. 38.99 ± 21.01 h, *p* < 0.001), and shorter postoperative hospital stay (7.76 ± 1.57 vs. 9.77 ± 3.80 days, *p* < 0.001). They also had superior preoperative 6MWD (448.60 ± 103.65 vs. 362.43 ± 91.85 m, *p* < 0.001), higher preoperative caloric (25.21 ± 6.33 vs. 16.34 ± 4.44 Kcal/kg IBW, *p* < 0.001) and protein intake (1.24 ± 0.35 vs. 0.62 ± 0.25 g/kg IBW, *p* < 0.001), higher discharge QoR-40c scores (*p* < 0.05), and lower Hospital Anxiety and Depression Scale (anxiety: *p* = 0.026, depression: *p* < 0.001) and Fatigue Severity Scale scores (*p* = 0.003).

**Conclusion:**

Multimodal prehabilitation (integrating exercise, nutrition, and psychological support) significantly improves perioperative outcomes in gastric cancer patients—including reducing postoperative complications, accelerating functional recovery, and enhancing psychological well-being.

**Clinical trial registration:**

ClinicalTrials.gov identifier ChiCTR2200062938.

## Introduction

Gastric cancer remains a critical global health challenge. In 2020, it was the fifth most common malignancy worldwide, with approximately 1.09 million new cases (5.6% of all cancers), and the fourth leading cause of cancer-related deaths, claiming approximately 770,000 lives (7.7% of total cancer mortality) (1, 2). This disease has a disproportionate impact on East Asian populations, shouldering 60% of the global burden. In 2022 alone, China reported 358,700 new cases, where gastric cancer ranks fifth in incidence and third in mortality among all malignancies (3–5). Surgical resection remains the fundamental curative approach for early-stage gastric cancer (6). For advanced cases, the emerging neoadjuvant therapies that combine chemotherapy with immunotherapy or targeted agents have shown potential in facilitating conversion surgery (7, 8). Nevertheless, attaining long-term tumor-free survival necessitates not only effective cancer control but also an improvement in perioperative resilience. A major challenge is to enhance patients’ tolerance to surgical stress and expedite postoperative recovery, an area that calls for innovative solutions from surgical teams.

The concept of prehabilitation, first put forward in 1946, has undergone substantial development with the introduction of Enhanced Recovery After Surgery (ERAS) protocols (9). In the context of global healthcare reforms that emphasize cost-effectiveness and patient-centered care, ERAS frameworks are in line with the increasing demand for accelerated recovery and reduced utilization of hospital resources (10, 11). Multimodal prehabilitation, which integrates exercise training, nutritional optimization, and psychological support, has emerged as a crucial part of ERAS by preparing patients both physically and mentally for surgical challenges (12). Growing evidence indicates that such interventions can reduce complication rates by 15–30%, shorten hospital stays by 2–4 days, improve patient-reported outcomes, and prove to be cost-effective (13).

Although prior studies have confirmed the value of single-component prehabilitation for gastric cancer patients, the efficacy of multimodal prehabilitation (integrating exercise, nutrition, and psychological support)—especially its tailored application to gastric cancer’s unique challenges and its impact on patient-reported outcomes (anxiety, fatigue, quality of life)—remains insufficiently studied. Gastric cancer patients face three interrelated perioperative barriers that justify the multimodal structure of our program: ① preoperative functional decline impairs surgical tolerance and predicts higher postoperative complications (14); ② malnutrition is associated with increased postoperative mortality and hospitalization costs (15); ③ preoperative anxiety/depression correlates with prolonged hospital stays and worse quality of life (16, 17). Thus, our program integrated exercise (to target functional decline), nutrition (to correct malnutrition), and psychological support (to alleviate emotional distress)—each component addressing a distinct, evidence-backed challenge. Notably, for nutritional optimization, we selected Suyisu (a tumor-targeted immunonutritional supplement) to address cancer-specific immune and metabolic derangements, as supported by international perioperative nutrition guidelines (18, 19). This prospective cohort study systematically assesses the influence of a structured prehabilitation program on perioperative functional capacity, postoperative recovery indicators, and complication prevention in patients undergoing radical gastrectomy. By analyzing multidimensional outcomes, including quality of life, psychological status, and rehabilitation trajectories, our aim is to establish an evidence-based management model to optimize the recovery process in gastric cancer surgery.

## Materials and methods

### Study design, trial registration, and ethics approval

This single-center, prospective randomized controlled trial (RCT) was conducted at the Department of Gastrointestinal Surgery, First Affiliated Hospital of Guangxi Medical University, from July 2022 to July 2024. The trial was registered at ClinicalTrials.gov (ID: ChiCTR2200062938) and approved by the Institutional Review Board of the hospital (no. 2022-KY-E-251). All enrolled patients provided written informed consent in accordance with the Declaration of Helsinki.

### Participants

Inclusion criteria: ① Histologically confirmed gastric adenocarcinoma; ② Planned D2 radical gastrectomy; ③ Age ranging from 18 to 75 years; ④ Adequate functional capacity, defined as the ability to walk more than 200 meters (trained research nurses accompanied each candidate to a standardized 200-meter indoor corridor marked with distance indicators and observed them completing the walk) or climb two flights of stairs without rest; ⑤ Absence of cognitive or psychiatric disorders.

Exclusion criteria: ① Previous neoadjuvant therapy; ② Comorbid malignancies; ③ Musculoskeletal limitations that contraindicate exercise; ④ Severe cardiopulmonary dysfunction (defined as (a) New York Heart Association Class IV heart failure; (b) Forced expiratory volume in 1 s < 30% of predicted value; or (c) Uncontrolled arrhythmia requiring urgent intervention), renal dysfunction (estimated glomerular filtration rate < 30 mL/min/1.73m^2^), or anemia (preoperative hemoglobin concentration < 60 g/L).

All patients in the prehabilitation and control groups received identical standardized anaesthesia and perioperative analgesia.

### Interventions

#### Prehabilitation group

Received a 1-week multimodal program plus standard perioperative care:

(1) Exercise: Aerobic training, progressive resistance training (targeting major muscle groups), flexibility routines, and inspiratory muscle training (using threshold-loading devices); 3 supervised sessions per week, 40 min per session, with intensity controlled at 60–80% of peak heart rate (monitored via telemetry).

(2) Nutrition: All patients underwent NRS-2002 screening. For patients with NRS-2002 ≥ 3, PG-SGA assessment, anthropometric measurement, and biochemical testing were performed. Nutritional support included whey protein supplementation (0.4 g/kg, administered post-exercise or pre-sleep) and Suyisu (a tumor-targeted immunonutritional supplement, 2 bottles/day for ≥7 days); the target intake was 25–30 kcal/kg/day (calories) and 1.2–1.5 g/kg/day (protein).

(3) Psychological support: 30-min individualized counseling sessions (provided by trained psychologists) twice weekly; baseline assessment using the Hospital Anxiety and Depression Scale (HADS) and Fatigue Severity Scale (FFS) plus cognitive restructuring.

#### Control group

Received standard perioperative care (routine preoperative education; no structured exercise, nutritional, or psychological intervention).

### Outcome measures

#### Primary endpoint

Overall incidence of postoperative complications within 30 days after radical gastrectomy. All complications were classified and recorded using the Clavien-Dindo system; included events were pulmonary complications (e.g., pneumonia, atelectasis), surgical site infections, gastrointestinal events (e.g., anastomotic leakage, ileus, transient nausea/vomiting), cardiovascular events, and other clinically relevant adverse events (e.g., low-grade fever, urinary tract infection).

#### Secondary endpoints

① Functional capacity: Evaluated by the 6-min walk distance (6MWD) and pulmonary function parameters (forced vital capacity [FVC], forced expiratory volume in one second [FEV1], peak expiratory flow [PEF]);
② Patient-reported outcomes: Assessed using the Chinese version of Quality of Recovery-40 (QoR-40c) (20), HADS, and FFS;
③ Thirty-day emergency visits or readmissions;
④ Physical performance: Measured via the 5-time sit-to-stand test and grip strength;
⑤ Metabolic and nutritional biomarkers: C-reactive protein (CRP), interleukin-6 (IL-6), CD4^+^/CD8^+^ ratio, albumin, and prealbumin;
⑥ Surgical outcomes: Time to ambulation, time to first flatus, and length of postoperative hospital stay;
⑦ Hospitalization costs: Defined as total in-hospital medical expenses from admission to discharge (including routine hospitalization costs: bed, nursing, surgery, intraoperative supplies, routine exams, basic medications; prehabilitation-related allied health service costs: physiotherapist exercise guidance, psychologist counseling, nutritional assessment, Suyisu supplements; postoperative recovery costs). Non-treatment expenses (patient meals, family accommodation) and out-of-hospital services (pre-admission outpatient exams) were excluded; the same counting standard was applied to both groups.

### Randomization and allocation concealment

Eligible patients were assigned to the prehabilitation group or control group via stratified block randomization. An independent biostatistician (not involved in recruitment) generated a random number sequence using SPSS 26.0 software with a block size of 4. Allocation was concealed using sequentially numbered, opaque sealed envelopes; after obtaining informed consent, an independent research nurse opened the next envelope to confirm group assignment.

### Blinding

Due to the nature of the multimodal intervention (exercise, counseling), patients and research nurses implementing the intervention could not be blinded. However, outcome assessors (e.g., staff measuring 6MWD, scoring QoR-40c/FFS) and statisticians (data analysis) were blinded to group allocation.

### Sample size calculation

Sample size was determined based on the primary endpoint (postoperative complications):

(1) Assumptions: 30% complication rate in the control group, 12% in the prehabilitation group; *α* = 0.05 (two-sided), power = 80%;
(2) Calculation: A minimum of 62 patients per group was required (calculated using G*Power 3.1). To account for 20% potential dropouts, 75 patients were randomized per group (total 150).

### Statistical analysis

All statistical analyses in this study were performed using SPSS 4.0, and all statistical graphs were generated using GraphPad 8.1. For two groups of continuous data, hypothesis testing was carried out using the Student’s t-test after verifying normality, independence, and homogeneity of variance. For multiple groups of continuous data, analysis of variance (ANOVA) was applied after confirming normality, independence, and homogeneity of variance. Correlation tests and comparisons of proportions for categorical data were conducted using the Chi-square test or Fisher’s exact test. Continuous data with a skewed distribution were described using the median and interquartile range, and group differences were assessed using the Mann–Whitney U test. In this study, a *P*-value of less than 0.05 was considered statistically significant.

## Results

### Baseline information

From July 2022 to July 2024, consecutive patients scheduled for radical gastrectomy were screened per predefined inclusion and exclusion criteria. A total of 150 patients were randomly allocated to this study, and 19 patients dropped out before completing the trial, resulting in 131 patients who finished the clinical trials (66 gastric cancer patients in the prehabilitation group and 65 in the control group). The reasons for dropout were categorized as follows: (1) Withdrawal of informed consent (*n* = 8): 5 patients voluntarily chose to transfer to other hospitals for surgical treatment, and 3 patients withdrew due to family concerns about the 1-week prehabilitation intervention duration, requesting early surgery; (2) Failure to complete the intervention (*n* = 11): 7 patients required urgent surgical intervention, and 4 patients experienced preoperative exacerbation of comorbidities (Supplementary Figure S1).

The average preoperative intervention duration in the prehabilitation group was 7.33 ± 1.00 days. The detailed baseline clinical characteristics of both groups are presented in Table 1. There were no statistically significant differences between the two groups in terms of gender, age, education level, comorbidities, smoking history, or TNM staging (all *p* > 0.05). Regarding surgical -related data, including Borrmann classification, surgical method, extent of resection, surgical duration, intraoperative blood loss, and anesthesia ASA score, no significant disparities were found between the two groups (all *p* > 0.05). Moreover, preoperative nutritional status indicators, such as BMI, NRS2002 score, PG-SGA score, hemoglobin, albumin, prealbumin, transferrin, daily caloric intake, and protein intake, also showed no significant differences between the two groups (all *p* > 0.05). These findings suggest that the two groups had comparable preoperative health statuses, treatment approaches, and nutritional conditions.

**Table 1:** Baseline comparison between the multi-mode pre-rehabilitation group and control group.

Characteristic	Pre-rehabilitation (*n* = 66)	Control (*n* = 65)	*P*
Sex (male/female)	40/26	46/19	0.221
Age (years)	55.92 ± 11.05	57.86 ± 8.67	0.267
NRS2002 score (<3/≥3)	46/20	45/20	0.954
PG-SGA score (<4/≥4)	30/36	27/38	0.651
BMI (kg/m2) (≤24/>24)	50/16	44/21	0.305
Hemoglobin (g/L)	121.67 ± 22.53	121.10 ± 21.19	0.881
Albumin (g/L)	38.17 ± 4.81	37.49 ± 5.54	0.452
Prealbumin (mg/L)	242.72 ± 57.45	231.74 ± 59.08	0.283
Transferrin (g/L)	2.20 ± 0.32	2.13 ± 0.37	0.282
Daily caloric intake (Kcal/kg IBW.d)	18.47 ± 5.53	16.99 ± 5.41	0.123
Protein intake (g/kg IBW.d)	0.75 ± 0.30	0.68 ± 0.28	0.170
Education: Primary school/junior high school/senior high school/junior college and above	16/31/14/5	24/22/14/5	0.373
Underlying disease (none/yes)	41/11	46/10	0.493
Smoking history (no/quit/Smoking)	45/11/10	35/17/13	0.232
Anesthesia ASA score (II/III)	49/17	51/14	0.524
TNM stag (I/II/III/IV)	21/15/29/1	23/17/22/3	0.538
Borrmann classification (I/II/III/IV)	7/15/39/5	6/19/33/7	0.712
Surgical method (endoscopic/robotic/open)	43/14/9	51/12/2	0.071
Resection range (proximal/distal/whole stomach)	2/56/8	6/51/8	0.329
Operation time (min)	298.27 ± 70.35	314.18 ± 101.21	0.268
Intraoperative blood loss (ml)	160.48 ± 131.34	200.08 ± 184.41	0.159
Clinical outcome			
First exhaust time (h)	58.32 ± 26.82	89.55 ± 26.14	<0.001
First feeding time (h)	48.43 ± 31.98	105.85 ± 57.36	<0.001
First time out of bed (h)	25.14 ± 10.63	38.99 ± 21.01	<0.001
Catheter removal time (days)	1.11 ± 0.36	1.35 ± 1.50	0.195
Gastric tube removal time (days)	1.92 ± 1.32	3.86 ± 2.33	<0.001
Postoperative pulmonary complications (none/yes)	62/4(6.1%)	51/14(21.5%)	0.012
30 days emergency department visits (none/yes)	63/3(4.5%)	57/8(12.3%)	0.109
30 days readmission (none/yes)	63/3(4.5%)	63/2(3.1%)	0.661
Total hospitalization days	16.38 ± 2.245	14.74 ± 4.24	0.006
Postoperative hospital stay	7.76 ± 1.57	9.77 ± 3.80	<0.001
Hospitalization expenses (Yuan)	68694.79 ± 15521.77	75611.64 ± 29618.99	0.096

PG-SGA (Patient-Generated Subjective Global Assessment) is a tool used to evaluate patients’ nutritional status; NRS2002 (Nutritional Risk Screening 2002) is a tool for assessing patients’ nutritional risks; BMI (Body Mass Index) = weight (kg)/height (m)^2^; IBW refers to the ideal body weight in kilograms, and the calculation formula is: IBW (kg) = 50 (for males) or 45.5 (for females) + 0.91 * [height (cm) − 152.4]; Borrmann classification is a histological morphological classification for gastric cancer; the ASA physical status classification system is a pre-anesthesia evaluation system established by the American Society of Anesthesiologists.

In terms of clinical outcomes, the prehabilitation group demonstrated more favorable recovery metrics compared to the control group. Specifically, they had an earlier time to first flatus (58.32 ± 26.82 vs. 89.55 ± 26.14 h, *p* < 0.001), a shorter interval to oral intake (48.43 ± 31.98 vs. 105.85 ± 57.36 h, *p* < 0.001), a reduced time to ambulation (25.14 ± 10.63 vs. 38.99 ± 21.01 h, *p* < 0.001), and an earlier nasogastric tube removal (1.92 ± 1.32 vs. 3.86 ± 2.33 days, *p* < 0.001). Additionally, the intervention group had significantly shorter postoperative hospital stays (7.76 ± 1.57 vs. 9.77 ± 3.80 days, *p* < 0.001), indicating enhanced recovery through multimodal prehabilitation. Although the hospitalization costs were lower in the prehabilitation group (¥68,694.79 ± 15,521.77 vs. ¥75,611.64 ± 29,618.99), this difference did not reach statistical significance (*p* = 0.096). No significant differences were observed in 30-day readmission rates or emergency department visits between the groups.

### Comparison of postoperative complications

As shown in Table 2, the prehabilitation group exhibited a significantly lower overall postoperative complication rate compared to the control group (9.1% [6/66] vs. 26.2% [17/65], *p* = 0.010). Subgroup analysis of Clavien-Dindo grades revealed that the between-group difference was mainly driven by Grade II complications: the prehabilitation group had a notably lower incidence of Grade II complications (4.5% [3/66] vs. 18.5% [12/65], *p* = 0.012), while no statistically significant differences were observed in Grade I (3.0% vs. 1.5%, *p* = 0.659), Grade III (1.5% vs. 4.6%, *p* = 0.617), Grade IV (0.0% vs. 1.5%, *p* = 0.498), or Grade V (0.0% vs. 0.0%, *p* = 1.000) complications. Among all complications, pulmonary infections (a major component of Grade II complications) showed the most remarkable reduction in the prehabilitation group (6.1% vs. 21.5%, *p* < 0.05), consistent with the improved preoperative pulmonary function observed in the intervention group.

**Table 2:** Comparison of postoperative complications between the multi-modal pre-rehabilitation group and the control group.

Clavien-Dindo grade	Pre-rehabilitation (*n* = 66)	Control (*n* = 65)	*P*
Total postoperative complications	6(9.1%)	17(26.2%)	0.010
Grade I	2(3.0%)	1(1.5%)	0.659
Grade II	3(4.5%)	12(18.5%)	0.012
Grade III	1(1.5%)	3(4.6%)	0.617
Grade IV	0(0.0%)	1(1.5%)	0.498
Grade V	0(0.0%)	0(0.0%)	1.000

Grade I: Mild self-limiting complications requiring only routine supportive care (e.g., oral medications, local wound care); Grade II: Complications requiring pharmacological intervention (excluding routine Grade I medications) or IV fluid support; Grade III: Complications requiring surgical, endoscopic, or radiological intervention; Grade IV: Life-threatening complications requiring ICU admission; Grade V: Death within 30 days after surgery.

### Impact of multimodal prehabilitation on physical capacity

**Figure 1:**
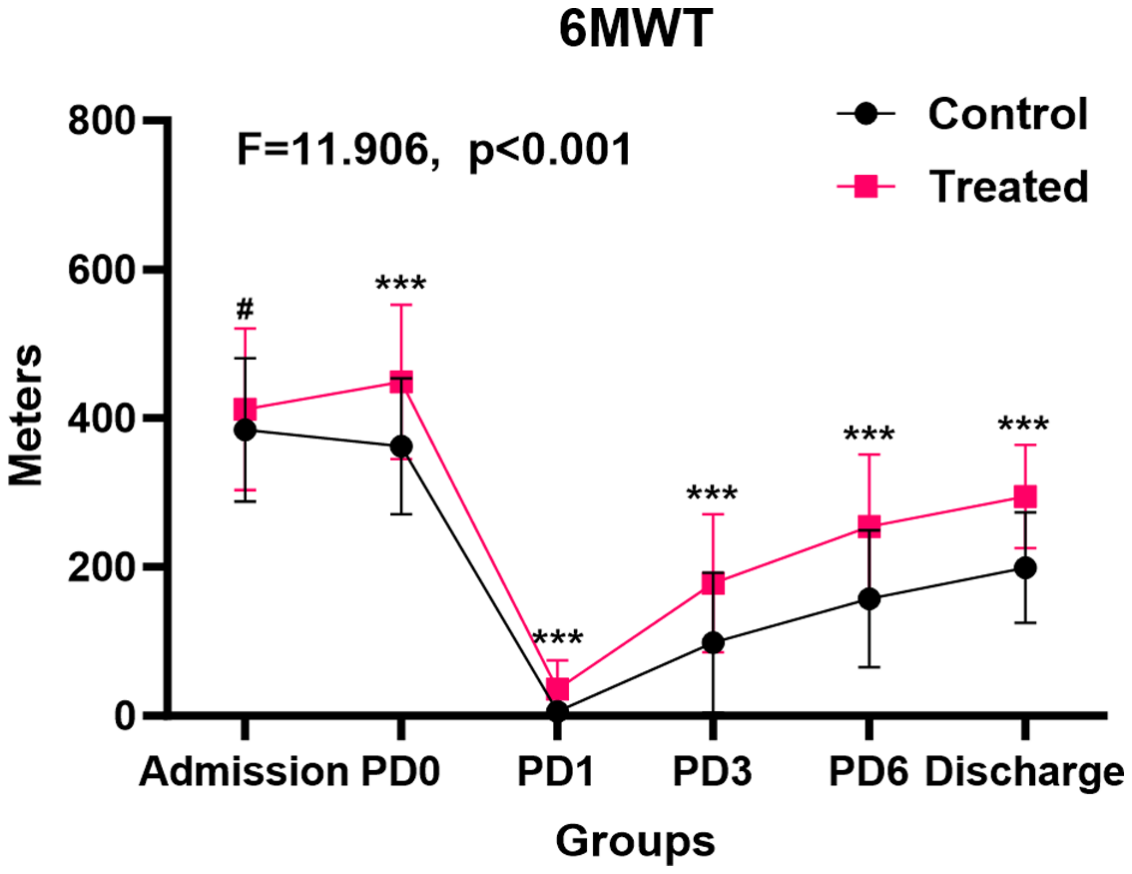
Comparison of the 6-min walk test during the perioperative period between the multimodal prehabilitation group and the treated group. # *p* > 0.05; * *p* < 0.05; ** *p* < 0.01, *** *p* < 0.001.

**Figure 2:**
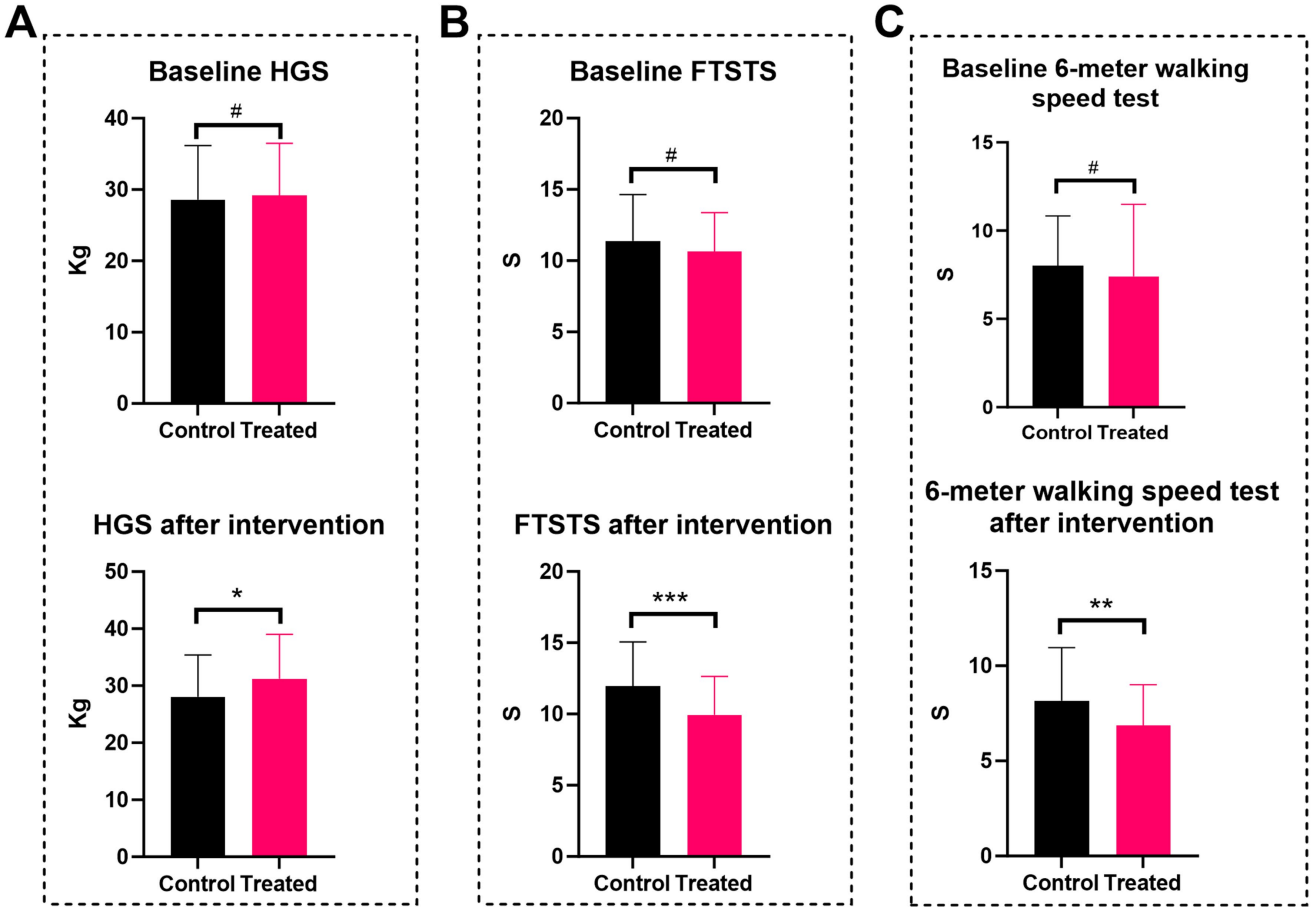
Comparison of physical fitness between patients in the multimodal prehabilitation group and the control group. **(A)** HGS; **(B)** FTSTS; **(C)** 6-meter walking speed test. # *p* > 0.05; * *p* < 0.05; ** *p* < 0.01, *** *p* < 0.001.

The 6-min walk test (6MWT) was serially evaluated at admission, preoperatively, and on postoperative days 1, 3, 6, and at discharge. Additionally, grip strength, 6-meter gait speed, and 5-time sit-to-stand tests were conducted preoperatively. At baseline, there was no inter-group difference in 6MWT distances (prehabilitation: 411.70 ± 108.97 m vs. control: 384.40 ± 96.41 m, *p* = 0.132). However, the prehabilitation group showed superior performance in subsequent assessments: on the preoperative day (448.60 ± 103.65 vs. 362.43 ± 91.85 m), postoperative day 1 (35.83 ± 38.41 vs. 5.88 ± 13.46 m), day 3 (178.23 ± 92.85 vs. 98.68 ± 94.85 m), day 6 (254.18 ± 96.88 vs. 157.60 ± 92.35 m), and at discharge (294.79 ± 69.50 vs. 198.80 ± 74.52 m; all *p* < 0.001) (Table 3). Repeated-measures ANOVA confirmed significant temporal improvements in 6MWT favoring the prehabilitation group (*F* = 11.906, *p* < 0.001; Figure 1). Although baseline grip strength, gait speed, and sit-to-stand performance were comparable between the groups, prehabilitation significantly enhanced these metrics after the intervention (all *p* < 0.05; Figures 2A–C), suggesting improved functional capacity.

**Table 3:** Changes in 6MWT (6-minute walk test) at various time points in both groups.

Group	Admission	Preoperative	PD1	PD3	PD6	Discharge
Pre-rehabilitation	411.70 ± 108.97	448.60 ± 103.65	35.83 ± 38.41	178.23 ± 92.85	254.18 ± 96.88	294.79 ± 69.50
Control	384.40 ± 96.41	362.43 ± 91.85	5.88 ± 13.46	98.68 ± 94.85	157.60 ± 92.35	198.80 ± 74.52
*p* value	0.132	<0.001	<0.001	<0.001	<0.001	<0.001

PD, postoperative days.

### Pulmonary function outcomes

**Figure 3:**
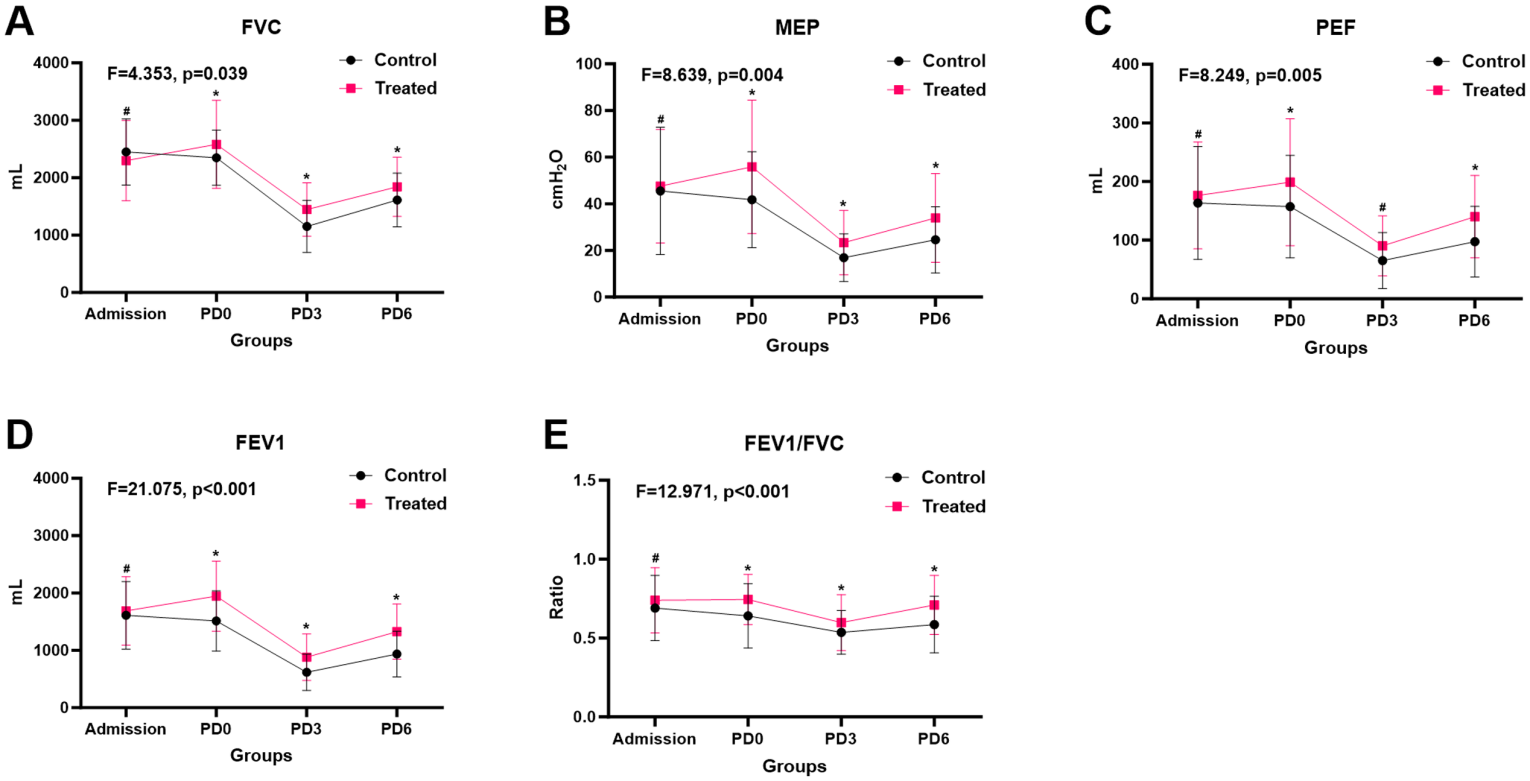
Changes in respiratory function at different time points among patients in the multimodal prehabilitation group and the control group. **(A)** FVC; **(B)** MEP; **(C)** PEF; **(D)** FEV1; **(E)** FEV1/FVC. # *p* > 0.05; * *p* < 0.05; ** *p* < 0.01, *** *p* < 0.001.

There were no baseline differences in pulmonary parameters (FVC, MEP, PEF, FEV1, FEV1/FVC). Compared to the control group, prehabilitation significantly increased preoperative FVC (*p* = 0.003), MEP (*p* = 0.001), and FEV1 (*p* < 0.001) (Supplementary Table S1). Longitudinal analysis revealed that the intervention group had superior postoperative pulmonary recovery across all metrics: FVC (*F* = 4.353, *p* = 0.039), MEP (*F* = 8.639, *p* = 0.004), PEF (*F* = 8.249, *p* = 0.005), FEV1 (*F* = 21.075, *p* < 0.001), and FEV1/FVC (*F* = 12.971, *p* < 0.001) (Figure 3).

### Nutritional status

**Figure 4:**
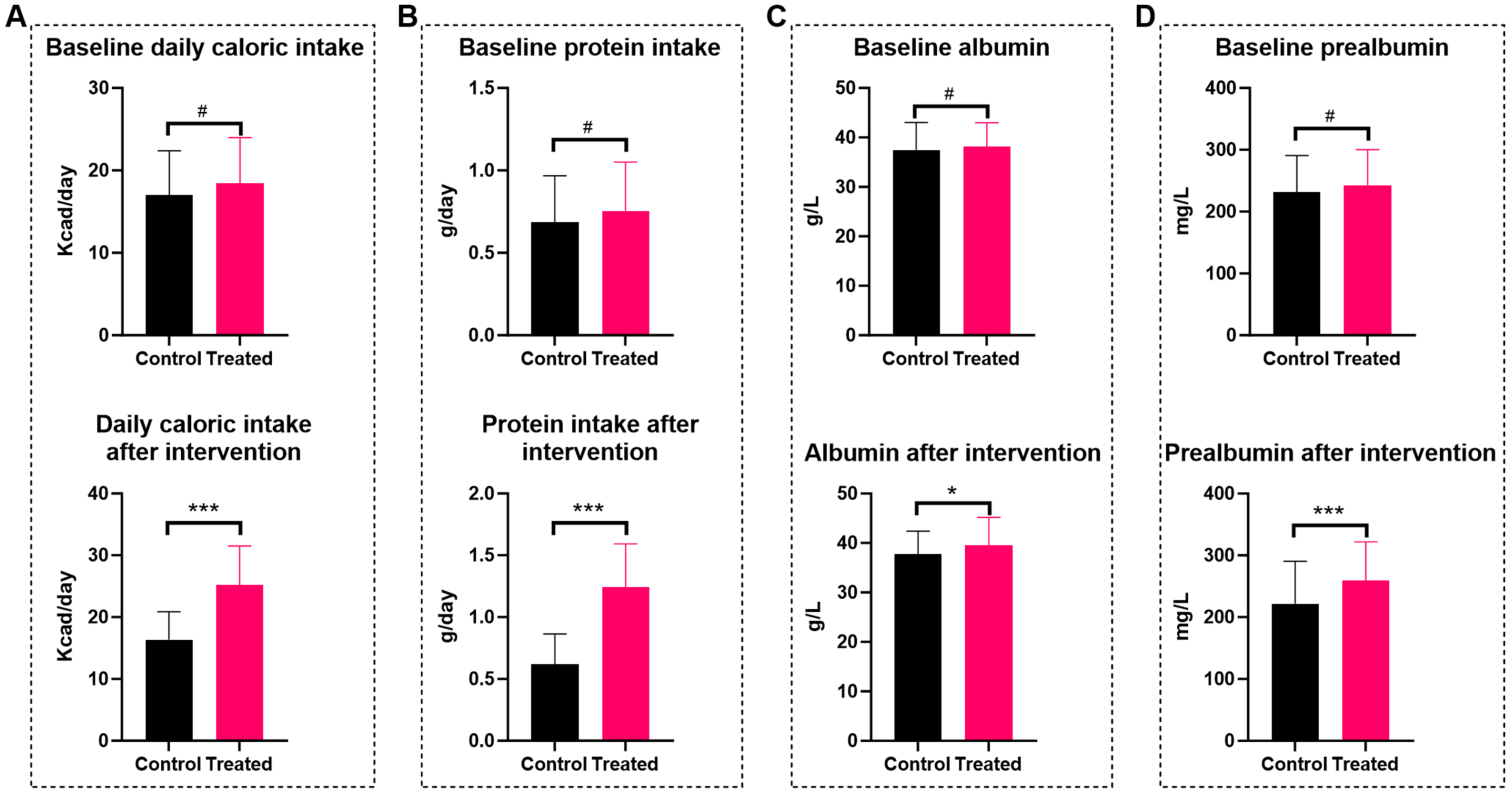
Comparison of nutritional status between patients in the multimodal prehabilitation group and the control group. **(A)** Daily caloric intake; **(B)** Protein intake; **(C)** Albumin; **(D)** Prealbumin. # *p* > 0.05; * *p* < 0.05; ** *p* < 0.01, *** *p* < 0.001.

At admission, there were no significant differences in baseline daily caloric intake (18.47 ± 5.53 vs. 16.99 ± 5.41 Kcal/kg IBW, *p* = 0.123) or daily protein intake (0.75 ± 0.30 vs. 0.68 ± 0.28 g/kg IBW, *p* = 0.170) between the prehabilitation and control groups. However, after approximately one week of preoperative prehabilitation, the prehabilitation group had significantly higher daily caloric intake (25.21 ± 6.33 vs. 16.34 ± 4.44 Kcal/kg IBW, *p* < 0.001) and daily protein intake (1.24 ± 0.35 vs. 0.62 ± 0.25 g/kg IBW, *p* < 0.001) compared to the control group (Figures 4A,B). Additionally, preoperative serum albumin and prealbumin levels were significantly higher in the prehabilitation group than in the control group, indicating that prehabilitation improves preoperative nutritional status (Figures 4C,D).

### Immune function

Lymphocyte subsets were measured at admission, pre-surgery, postoperative day 1, and day 3. At admission, the levels of CD4 + T cells, CD8 + T cells, and the CD4+/CD8 + ratio were similar between the two groups, suggesting comparable immune statuses at baseline. Repeated measures ANOVA comparing immune status across the perioperative period showed no significant differences between the prehabilitation and control groups in CD4 + T cells (*F* = 0.084, *p* = 0.772), CD8 + T cells (*F* = 0.002, *p* = 0.967), or the CD4+/CD8 + ratio (*F* = 1.003, *p* = 0.319), implying that prehabilitation has little effect on lymphocyte subsets (Supplementary Figure S1).

### Inflammatory response and insulin resistance

There were no statistically significant differences in interleukin-6 (IL-6) and C-reactive protein (CRP) levels between the two groups at any time point. Repeated measures ANOVA indicated that the differences in CRP (*F* = 0.505, *p* = 0.479) and IL-6 (*F* = 2.960, *p* = 0.088) between the intervention and control groups were also not statistically significant (Supplementary Figures S2A,B). Although there were no significant differences observed at any time point, the insulin resistance index in the prehabilitation group was lower than that in the control group on postoperative day 1 and day 3. However, repeated measures ANOVA showed no statistically significant difference in the insulin resistance index between the intervention and control groups (*F* = 0.143, *p* = 0.706) (Supplementary Figure S2C).

### QoR-40c scores

**Figure 5:**
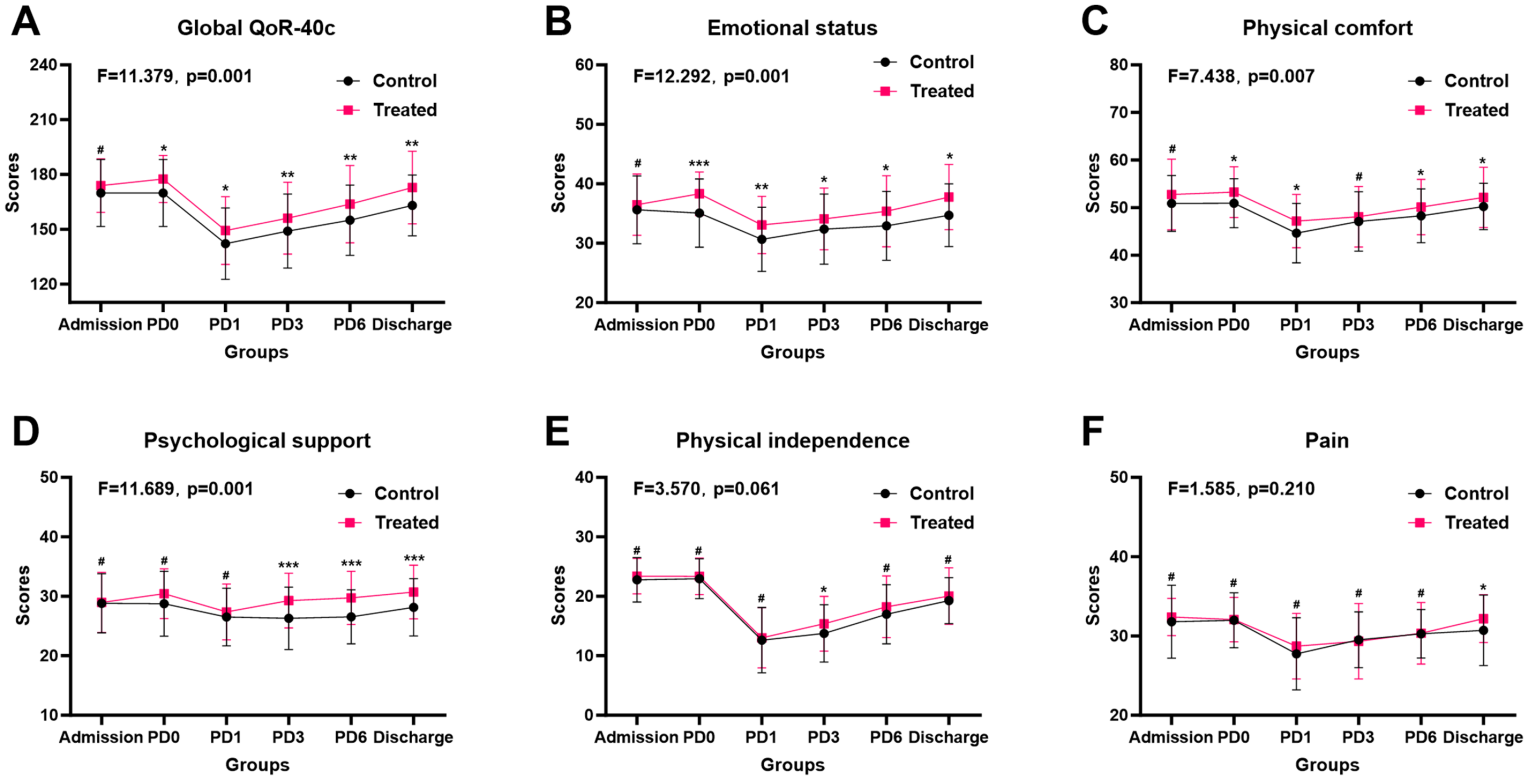
Comparison of perioperative quality of life between patients in the multimodal prehabilitation group and those in the control group. **(A)** Global QoR-40c; **(B)** Emotional status; **(C)** Physical comfort; **(D)** Psychological; **(E)** Physical independence; **(F)** Pain. # *p* > 0.05; * *p* < 0.05; ** *p* < 0.01, *** *p* < 0.001.

The distribution of the total QoR-40c scores over the perioperative period is depicted in Figure 5A. There was no significant difference in the total QoR-40c scores between the two groups at admission. However, patients in the multimodal prehabilitation group had significantly higher scores than those in the control group on the day before surgery, postoperative day 1, day 3, day 6, and at discharge (all *p* < 0.05). Repeated measures ANOVA also revealed that the overall quality of life scores for the prehabilitation group were significantly higher than those for the control group (*F* = 11.379, *p* = 0.001), suggesting that multimodal prehabilitation can significantly improve the quality of life during the perioperative period. Analyzing the five dimensions of the QoR-40c scale, patients in the prehabilitation group showed significant improvements in emotional state (*F* = 12.292, *p* = 0.001), physical comfort (*F* = 7.438, *p* = 0.007), and psychological support (*F* = 11.689, *p* = 0.001) following preoperative interventions (Figures 5B–F).

### Psychological outcomes

Changes in HADS scores for the multimodal prehabilitation group and the control group are presented in Supplementary Tables S2 and Supplementary Table S3. At admission, there were no statistically significant differences in HADS-A (anxiety) (*p* = 0.470) and HADS-D (depression) scores (*p* = 0.521) between the two groups. However, the multimodal prehabilitation group had significantly lower HADS-A and HADS-D scores than the control group at several time points: pre-surgery, postoperative day 3, postoperative day 6, and at discharge. Repeated measures ANOVA results indicated that multimodal prehabilitation significantly reduced anxiety (*F* = 5.073, *p* = 0.026) and depression (*F* = 14.209, *p* < 0.001) during the perioperative period.

### Fatigue severity

At admission, there were no statistically significant differences in FSS scores between the two groups (*p* = 0.606). However, after undergoing multimodal prehabilitation, the prehabilitation group exhibited significantly lower fatigue indices than the control group on the day before surgery (*p* = 0.026), postoperative day 3 (*p* = 0.030), postoperative day 6 (*p* = 0.003), and at discharge (*p* = 0.031). Additionally, repeated measures ANOVA results indicated a significant difference in fatigue levels between the two groups, with the intervention group performing better than the control group (*F* = 9.059, *p* = 0.003).

## Discussion

Gastrectomy remains the cornerstone of curative treatment for gastric cancer, yet postoperative complications, such as pulmonary infections and prolonged gastrointestinal dysfunction, and extended hospital stays continue to pose significant challenges to perioperative care, even with the broader adoption of ERAS protocols (21, 22). While ERAS has streamlined intraoperative and postoperative management, preoperative optimization—a critical link in the ERAS framework—has remained relatively underexplored in gastric cancer, particularly for multimodal prehabilitation that integrates exercise, nutrition, and psychological support. This gap is notable given that gastric cancer patients frequently face interconnected perioperative barriers—functional decline, malnutrition, and preoperative anxiety—that single-component interventions struggle to address, highlighting the need to contextualize new findings within existing research to advance clinical practice.

Existing literature on prehabilitation for gastrointestinal cancer has yielded inconsistent conclusions, with key gaps that limit its application to gastric cancer. For instance, van Rooijen et al. (23) conducted a multicenter trial in 714 colorectal cancer patients and demonstrated that multimodal prehabilitation improved functional capacity and reduced complications, but their 4-week intervention duration is impractical for gastric cancer. As noted in recent guidelines, gastric cancer often requires timely surgical intervention to avoid tumor progression, making prolonged prehabilitation courses difficult to implement in clinical settings. Conversely, Minnella et al. (14) explored exercise and nutrition prehabilitation in esophagogastric cancer and found improvements in functional capacity but no reduction in postoperative complications—likely due to the omission of psychological support. This oversight is critical for gastric cancer patients, as prior work by Lee et al. (15), and our own preliminary observations have shown that preoperative anxiety correlates with delayed ileus and longer hospital stays, underscoring the need for holistic interventions. Single-component nutritional support has also shown limitations: 30–40% of gastric cancer patients remain malnourished despite preoperative nutrition alone, as surgical stress exacerbates metabolic derangements that cannot be reversed by nutrition alone. Several randomized controlled trials (RCTs) and meta-analyses suggest that although prehabilitation may improve preoperative functional reserves, it does not have a significant impact on postoperative complications, length of hospital stay, or costs (14, 24–26). Moreover, a study on a 4-week pre-rehabilitation intervention in colorectal cancer patients suggested that pre-rehabilitation did not influence clinical outcomes (27). These inconsistencies and gaps in existing research underscore the need for a gastric cancer-specific multimodal prehabilitation strategy that balances efficacy with clinical feasibility.

Against this backdrop, the current trial’s findings advance the field by addressing these unmet needs through a 1-week short-course multimodal intervention, integrating exercise, tumor-targeted nutrition (Suyisu), and psychological support. Our results—showing a significant reduction in overall postoperative complications and shorter hospital stays—are particularly notable for their alignment with clinical realities: the 1-week duration avoids delaying surgery, a key concern for gastric cancer management, while still delivering meaningful perioperative benefits. The marked reduction in pulmonary infections further contextualizes these findings within ERAS guidelines: while ERAS for gastrectomy recommends respiratory care, it lacks specific evidence for preoperative inspiratory muscle training— a core component of our exercise intervention. Our data confirm that this training improves preoperative forced expiratory volume in one second and forced vital capacity, directly linking functional optimization to reduced pulmonary complications and filling an evidence gap in ERAS implementation for gastric cancer.

Beyond complication reduction, our findings also extend existing knowledge by highlighting the synergistic value of three interrelated components, rather than single modalities. For example, the prehabilitation group’s improved preoperative nutritional status—with higher daily caloric and protein intake—was paired with reduced anxiety and faster oral intake initiation. This aligns with Braga’s (19) hypothesis that nutritional support alone is insufficient to mitigate surgical stress, but adds new insight by demonstrating that combining nutrition with psychological support addresses the emotional barriers to early oral intake—a common challenge in gastric cancer recovery (28–30). Additionally, our focus on patient-reported outcomes (PROs) fills another gap in the literature: while most prior prehabilitation studies emphasize objective metrics like 6MWD, our data show significant improvements in QoR-40c scores and reduced fatigue, which align with Godino et al.’s call for more patient-centric outcomes in gastric cancer research (16, 17, 31). These PRO improvements are clinically relevant, as they reflect not just physical recovery, but also the psychological well-being that is critical for long-term adherence to postoperative care and quality of life.

This RCT systematically evaluated multimodal prehabilitation in gastric cancer patients, complying with CONSORT guidelines to ensure transparency. Consistent with our hypothesis, the intervention improved perioperative outcomes—likely due to synergistic effects of exercise (enhancing functional reserve), nutrition (correcting malnutrition), and psychological support (reducing anxiety).

### Limitations

① Single-center design may limit generalizability; ② Short follow-up (no long-term survival data); ③ No blinding of patients/intervention staff (potential performance bias, mitigated by blinded outcome assessors).

### Generalizability

The intervention is feasible for gastric cancer patients scheduled for D2 radical gastrectomy (1-week duration avoids delaying surgery, critical for fast-growing tumors). Future multicenter RCTs with longer follow-up are needed to validate long-term efficacy.

## Conclusion

This study evaluated the effects of multimodal prehabilitation in patients undergoing radical gastric cancer surgery by comparing various indicators between the control and prehabilitation groups. The results suggest that multimodal prehabilitation has a positive impact on physiological indicators and offers significant advantages in psychological well-being, pain management, the recovery process, and reducing complications. This provides new evidence for comprehensive rehabilitation in gastric cancer patients during the perioperative period. Future research should clarify the optimal implementation methods for multimodal prehabilitation and explore its long-term effects on quality of life.

## Data Availability

The original contributions presented in the study are included in the article/Supplementary material, further inquiries can be directed to the corresponding author.
