# Supplementary material for: Multimodal prehabilitation enhances perioperative outcomes in gastric cancer patients: a single-center randomized controlled trial
Source: Front Nutr. 2026 Feb 3;12:1676180. doi: 10.3389/fnut.2025.1676180 (PMC12909225; doi:10.3389/fnut.2025.1676180)
Supplement: Supplementary file 1 [file Table_1.docx]

##### **Figure S1.** CONSORT-compliant patient flow diagram.

##### **
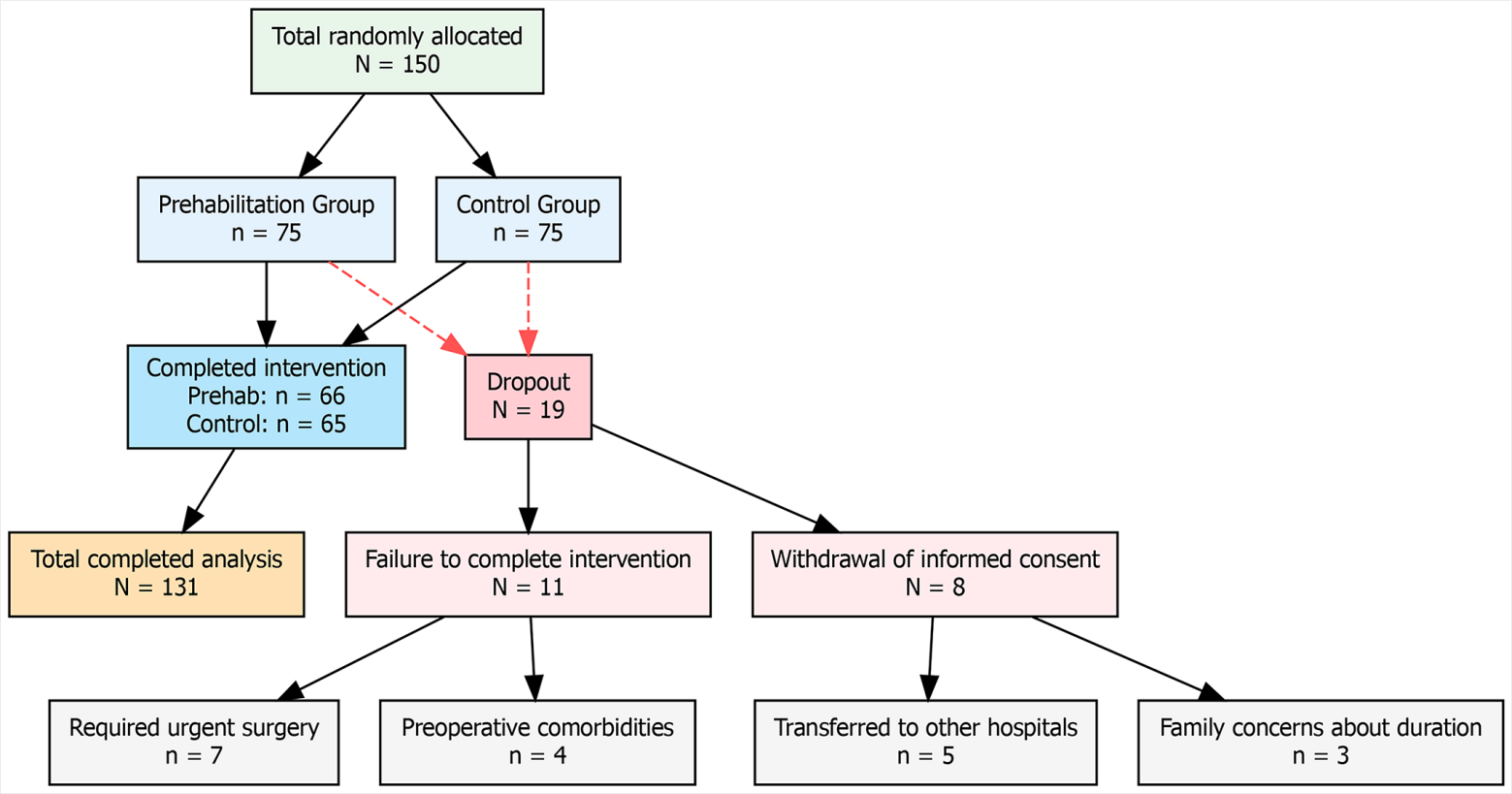
**

**Figure S2.** Changes in inflammation and insulin resistance among patients in the multimodal prehabilitation group and the control group.

**
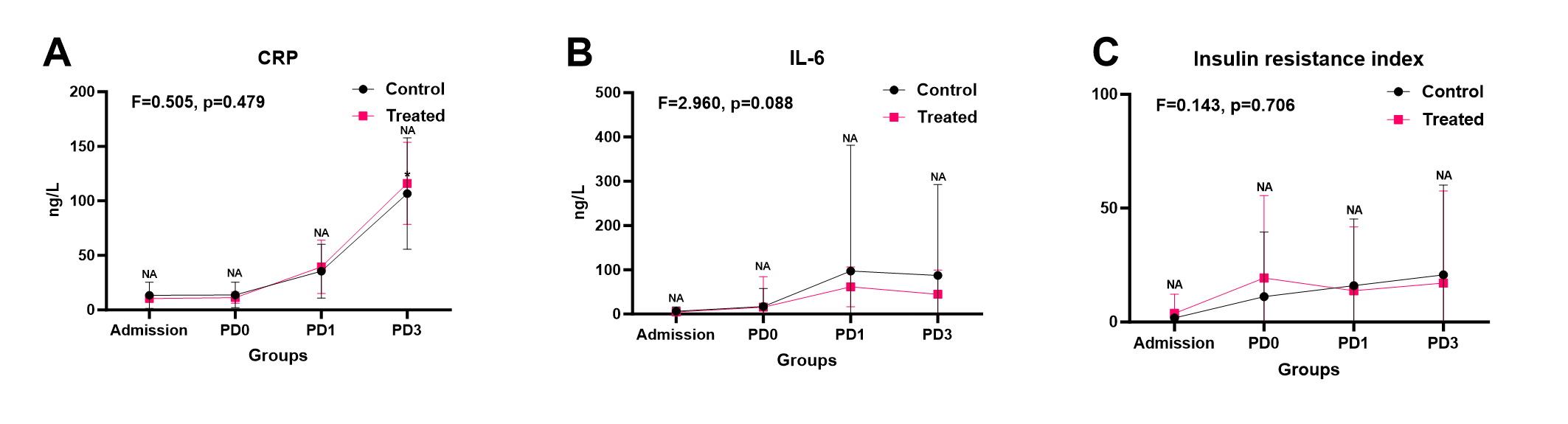
**

Notes: (A) CRP; (B) IL-6; (C) Insulin resistance index.

##### **Table S1.** Comparison of lung function before and after intervention between the prehabilitation group and the control group.

| Group | FVC(ml) | MEP(cmH2O) | PEF(L/min) | FEV1(ml) | FEV1/FVC |
| --- | --- | --- | --- | --- | --- |
| pre-intervention | 2373.25±644.33 | 46.53±25.83 | 169.86±93.77 | 1651.59±591.82 | 0.71±0.21 |
| post-intervention | 2465.94±651.21 | 48.86±25.88 | 178.37±100.48 | 1731.92±607.38 | 0.69±0.19 |
| *p* value | <0.001 | <0.001 | <0.001 | <0.001 | <0.001 |

##### **Table S2.** Changes in HADS-A Scores at Various Time Points.

| Group | Admission | Preoperative | PD1 | PD3 | PD6 | Discharge |
| --- | --- | --- | --- | --- | --- | --- |
| Pre-rehabilitation | 6.95±3.57 | 5.11±3.39 | 7.56±3.77 | 6.55±3.68 | 6.02±4.11 | 4.35±3.31 |
| Control | 6.45±4.44 | 6.91±4.94 | 7.88±4.36 | 8.15±4.24 | 7.83±3.88 | 6.11±3.61 |
| *p* value | 0.470 | 0.016 | 0.658 | 0.022 | 0.010 | 0.004 |

##### **Table S3.** Changes in HADS-D Scores at Various Time Points.

| Group | Admission | Preoperative | PD1 | PD3 | PD6 | Discharge |
| --- | --- | --- | --- | --- | --- | --- |
| Pre-rehabilitation | 5.83±3.37 | 4.52±3.33 | 7.20±4.42 | 6.36±3.87 | 5.88±4.28 | 4.45±3.63 |
| Control | 6.28±4.46 | 6.68±4.98 | 8.40±4.59 | 8.37±4.09 | 8.38±4.03 | 7.69±3.80 |
| *p* value | 0.521 | 0.004 | 0.129 | 0.005 | 0.001 | <0.001 |
